# 

**Published:** 1905-02-18

**Authors:** 


### NOTICE TO CORRESPONDENTS.

All MSS., Letters, Books for Review, and other matters intended for the Editor should be addressed The Editor, "The Hospital," The Hospital Buildings, 28 & 29 Southampton Street, Strand, W.C.

The Editor cannot undertake to return rejected MS., even when accompanied by stamped directed envelope.

**Notice to Advertisers and Subscribers.**

All Advertisements, Orders for Copies of The Hospital, and Business Communications should be addressed to **THE MANAGER (Not to the Editor),** The Hospital, 28 & 29 Southampton Street, Strand, London, W.C.

The Cost of Subscription, post free, is as follows:—Per week, 3½d.; per quarter, 3s. 3d.; per half-year, 6s. 6d.; per annum, 13s. Foreign and Colonial:—Per annum, 19s. 6d., payable, in all cases, in advance.

**The Monthly part for February is now ready. Price 1s., by post, 1s. 3d.**

### BOOKS RECEIVED.

J. Bale, Son and Danielsson, Limited.

"The Surgical Treatment of Facial Neuralgia." By J. Hutchinson, Jun., F.R.C.S.

Simpkin, Marshall, Hamilton, Kent and Co., Limited.

"The Plenum or Propulsion System of Heating and Ventilating." By Harold Griffiths.

Philip Wellby.

"The Annals of Psychical Science." No. 1. January 1905.

Baillière, Tindall and Cox.

"Autobiography of Frederick James Gant, F.R.C.S."

"The Preparation and After Treatment of Section Cases." By Stewart McKay, M.B., M.Ch.

"Cancer and its Treatment." By A. W. Mayo Robson, F.R.C.S.

"Dictionary of Medicine," Supplement. By George M. Gould, M.D.

J. and H. Bell, Limited, Nottingham.

"The Holy Communion: a Possible Source of Infection." By Hy. Blandy, L.D.S.

Henry J. Glaisher.

"A Short Essay on Insanity." By Chas. Williams, L.R.C.P.

Skeffington and Son.

"Religion *v.* Health." By Norman Porritt.

S. H. Benson.

"Force in Advertising."

T. Fisher Unwin.

"Catherine Furze." By Mark Rutherford.

Kegan Paul, Trench, Trübner, and Co.

"Ambidexterity." By J. Jackson, F.E.I.S.

The Scientific Press, Ltd.

"An Elementary Treatise on the Light Treatment for Nurses." By J. H. Sequira, M.D.

### BURDETT'S HOSPITALS AND CHARITIES 1905.

### An Indispensable Work of Reference.

THE PRESENT VOLUME CONTAINS—

A Ten Years' Review of the Expenditure of the London Hospitals.

A Ten Years' Review of the Free and Provident Dispensaries of London.

Reviews on the Nursing Department and its Cost, exhaustively treated in all its aspects.

Reviews on the Work and Cost of Hospitals, Missionary Societies, Orphanages, American and Colonial Hospitals, and other Subjects of Practical Interest.

### A DIRECTORY

of Information relating to Hospitals, Charities, and Allied Institutions throughout the English-speaking World.

**Over 1,000 pages, cloth gilt. 5s. 5d. post free.**

*Of all Booksellers, or of*

THE SCIENTIFIC PRESS, LTD.,

28 and 29 Southampton Street, Strand, London, W.C.

### MEDICAL APPOINTMENTS.

L. Bowman, M.B., C.M.Edin., Certifying Surgeon under the Factory and Workshop Act for the Cardenden District of the county of Fife. **Arthur H. Burnett**, M.R.C.S.Eng., L.R.C.P.Lond. House Surgeon to the Rochdale Infirmary. **J. W. Byers**, M.D., R.U.I., has been reappointed examiner in the Department of Midwifery in the Royal University of Ireland. **G. W. Kilner Crosland**, M.R.C.S., L.R.C.P.Lond., Honorary Surgeon to the Huddersfield Infirmary. **J. Sidney Pearson**, M.A.Cantab, M.R.C.S., L.R.C.P.Lond., Casualty Officer and House Surgeon to the Victoria Hospital for Children, Chelsea. **J. George Rowell**, M.R.C.S., L.R.C.P.Lond., Honorary Surgeon to the Huddersfield Infirmary. **Graham Scott**, M.R.C.S., L.R.C.P.London, Junior Anæsthetist to the Central London Throat and Ear Hospital. **David Shannon**, M.B., Ch.B., Clinical Assistant to the Chelsea Hospital for Women.

### Medical Appointments.

THE SERVICES of THREE DENTAL SURGEONS are required for duty with the Naval forces in the United Kingdom from April 1st next.

They will be required to devote their whole time to their duties and will receive an inclusive salary of £1 per diem, and, when necessary, travelling expenses. The period of engagement will be for one year and under conditions specified in the contract.

The necessary dental appliances will be provided.

The Dental Surgeons will be stationed at Portsmouth, Plymouth, and Chatham, but will be required to give attendance elsewhere as directed by the Naval Commander-in-Chief.

Application, by letter, should be addressed to—

THE DIRECTOR-GENERAL,
Medical Department of the Navy,
18 Victoria Street, London, S.W., not later than MARCH 7th, and should be accompanied by evidence of age and qualifications and by not more than two recent testimonials. (1323)

### A FEW PAGES
### . . FROM OUR . .
### CATALOGUE OF NURSING MANUALS.

### CATALOGUE
OF
### NURSING MANUALS
PUBLISHED BY
**The Scientific Press, Limited.**

### GENERAL.

**THE NURSING PROFESSION: HOW AND WHERE TO TRAIN.**
Being a Guide to Training for the Calling of a Nurse, with Particulars of Nurse Training Schools in the United Kingdom and Abroad, and an Outline of the Principal Laws affecting Nurses, &c.
Edited by Sir Henry Burdett, K.C.B.
Crown 8vo, cloth - - - - - 2/- net
2/4
Post free

**A HANDBOOK FOR NURSES.**
By J.K. Watson, M.D., M.B., C.M.
New and revised Edition. Crown 8vo, profusely illustrated, cloth gilt - - - - 5/- net
5/4
Post free

**NURSING: ITS THEORY AND PRACTICE.**
Being a complete Text-book of Medical, Surgical and Monthly Nursing.
By Percy G. Lewis, M.D., M.R.C.S., L.S.A.
New Edition, Enlarged and Revised (17th Thousand), profusely illustrated with over 100 new Cuts. Crown 8vo, cloth gilt - - - - - 3/6
Post free

28 and 29 Southampton Street, Strand, London, W.C.

NURSING MANUALS. 3

### GENERAL (*continued*).

**ELEMENTARY ANATOMY AND SURGERY FOR NURSES.**
By William McAdam Eccles, M.S. Lond., M.B., F.R.C.S., L.R.C.P.
Crown 8vo, profusely illustrated, cloth - - 2/6
Post free

**ELEMENTARY PHYSIOLOGY FOR NURSES.**
By C. F. Marshall, M.D., B.Sc., F.R.C.S.
Crown 8vo, illustrated, cloth - - - - 2/-
Post free

**ELEMENTS OF ANATOMY AND PHYSIOLOGY.**
By W. Bernard Secretan, M.D.
Crown 8vo, profusely illustrated, cloth - - 2/- net
2/3
Post free

**THE HUMAN BODY.** A Manual of Physiology and Personal Hygiene.
By B. P. Colton, Professor of Natural Science, Illinois University.
Crown 8vo, profusely illustrated, cloth gilt - 5/-
Post free

**SURGICAL WARD-WORK AND NURSING.**
By Alexander Miles, M.D. Edin., C.M., F.R.C.S.E.
Second Edition, Enlarged and entirely Revised.
Demy 8vo, copiously illustrated, cloth gilt 3/6 net
3/10
Post free

**HOSPITAL SISTERS AND THEIR DUTIES.**
By Eva C. E. Lückes, Matron, London Hospital.
Third Edition, enlarged and partly re-written, crown 8vo, about 200 pp., cloth gilt - - 2/6
Post free

28 and 29 Southampton Street, Strand, London, W.C.

NURSING MANUALS. 5

### SPECIAL TEXT-BOOKS.

**OPHTHALMIC NURSING.**
By Sydney Stephenson, M.B., F.R.C.S.E.
New and Revised Edition. Crown 8vo, profusely illustrated with Original Drawings, list of instruments required, and a Glossary of Terms, cloth - - - - 3/6 net
3/10
Post free

**NURSING IN DISEASES OF THE THROAT, NOSE AND EAR.**
By P. Macleod Yearsley, F.R.C.S. Eng., M.R.C.S., L.R.C.P. Lond.
Demy 8vo, illustrated, cloth - - - - 2/6
Post free

**ART OF MASSAGE.**
By A. Creighton Hale.
Second Edition, Demy 8vo, 150 pp., profusely illustrated with over 70 special Cuts, cloth gilt - - - - - - - - 6/-
Post free

**MEDICAL GYMNASTICS, INCLUDING THE SCHOTT (NAUHEIM) MOVEMENTS.**
Being a Text-book of Massage and Mechanical Therapeutics Generally.
By Axel V. Grafstrom, B.Sc., M.D.
Crown 8vo, illustrated, cloth - - - - 2/6
Post free

28 and 29 Southampton Street, Strand, London, W.C.

NURSING MANUALS. 7

### SPECIAL TEXT-BOOKS (*continued*).

**THE MODERN NURSING OF CONSUMPTION.**
By Dr. Jane H. Walker.
Crown 8vo, limp cloth - - - - - - 1/- net
1/2
Post free

**THE SCHOTT TREATMENT FOR CHRONIC HEART DISEASES.**
By Richard Greene, F.R.C.P. Ed.
Second Edition, demy 8vo, paper cover - - 6d.
Post free

### MIDWIFERY.

**A COMPLETE HANDBOOK OF MIDWIFERY.**

By J. K. Watson, M.D. Edin., Author of "A Handbook for Nurses."

Crown 8vo, over 350 pp., profusely illustrated with 143 Diagrams - - - - - 6/- net

6/4

Post free

**A PRACTICAL HANDBOOK OF MIDWIFERY.**
By Francis W. Haultain, M.D.
New and Revised Edition. 18mo, profusely illustrated with Original Cuts, Tables, etc.; handsomely bound in leather - - - 6/- net
6/3
Post free

**NURSING NOTES ON MIDWIFERY AND GYNÆCOLOGY.**
By Sister Ross, Rotunda Hospital, Dublin.
Demy 16mo, cloth - - - - - - - 2/- net
(Suitable size for apron pocket.) 2/1
Post free

**THE MIDWIVES' POCKET BOOK** (with Prefatory Chapter on the Midwives Bill).
By Honnor Morten.
Small crown 8vo, cloth - - - - - 1/-
Post free

The Scientific Press, Limited.

**A COMPLETE CATALOGUE POST FREE ON APPLICATION.**

**The Scientific Press, Ltd.,** 28 & 29 SOUTHAMPTON STREET, STRAND, LONDON, W.C.

